# Cell wall associated protein TasA provides an initial binding component to extracellular polysaccharides in dual-species biofilm

**DOI:** 10.1038/s41598-018-27548-1

**Published:** 2018-06-19

**Authors:** Danielle Duanis-Assaf, Tal Duanis-Assaf, Guanghong Zeng, Rikke Louise Meyer, Meital Reches, Doron Steinberg, Moshe Shemesh

**Affiliations:** 1 Department of Food Quality and Safety, Institute for Postharvest Technology and Food Sciences, Agricultural Research Organization (ARO), Volcani Center, Rishon LeZion, Israel; 2 Biofilm Research Laboratory, Institute of Dental Sciences, Faculty of Dental Medicine, Hebrew University-Hadassah, Jerusalem, Israel; 3 Institute of Chemistry, Hebrew University of Jerusalem, Jerusalem, Israel; 4 iNANO, Aarhus University, Aarhus C, Denmark

## Abstract

Many bacteria in biofilm surround themselves by an extracellular matrix composed mainly of extracellular polysaccharide (EP), proteins such as amyloid-like fibers (ALF) and nucleic acids. While the importance of EP in attachment and acceleration of biofilm by a number of different bacterial species is well established, the contribution of ALF to attachment in multispecies biofilm remains unknown. The study presented here aimed to investigate the role of TasA, a precursor for ALF, in cell-cell interactions in dual-species biofilms of *Bacillus subtilis* and *Streptococcus mutans*. Expression of major *B*. *subtilis* matrix operons was significantly up-regulated in the presence of *S*. *mutans* during different stages of biofilm formation, suggesting that the two species interacted and modulated gene expression in each other. Wild-type *B*. *subtilis* expressing TasA adhered strongly to *S*. *mutans* biofilm, while a TasA-deficient mutant was less adhesive and consequently less abundant in the dual-species biofilm. Dextran, a biofilm polysaccharide, induced aggregation of *B*. *subtilis* and stimulated adhesion to *S*. *mutans* biofilms. This effect was only observed in the wild-type strain, suggesting that interactions between TasA and dextran-associated EP plays an important role in inter-species interactions during initial stages of multispecies biofilm development.

## Introduction

Biofilm is defined as a complex structure of microorganisms adhering to each other and/or to surfaces, embedded in an extracellular matrix^1^. The variety of species and their relative abundance dictates the overall properties of the biofilm in terms of stress tolerance and virulence potential^2^.

The extracellular matrix, mostly produced by the microorganisms themselves, consists of different types of biopolymers that form the biofilm’s three-dimensional architecture, and is responsible for adhesion to surfaces and for cohesion within the biofilm^3^. The matrix mainly consists of polysaccharides, proteins, nucleic acids and lipids^1^.

In many bacteria, extracellular polysaccharides (EP) are indispensable for biofilm formation^4,5^. Moreover, in multispecies biofilms the presence of species that produce EP may lead to the integration of other species even if they do not synthesize matrix polymers themselves^6^.

Recent studies have shown that many biofilm-producing microorganisms can produce amyloid-like fibers (ALF)^7^. Among these microorganisms are *Bacillus subtilis* (Romero, Aguilar *et al*.^22^), *Streptococcus mutans* (Oli, Otoo *et al*. 2012), *Escherichia coli*^8,9^, *Staphylococcus aureus*^9^ and *Pseudomonas aeruginosa*^10^. Although the role of ALF in mono-species biofilm formation has been studied before, its role in multispecies microbial communities remains unclear^11^.

The dental biofilm is a classic example of diverse, complex multispecies biofilm. It appears that one of the most predominant class in the oral biofilm is bacilli^12^. Both, *B*. *subtilis* and *S*. *mutans* belong to the class of bacilli and are used as model organisms for studying biofilm formation. While, *S*. *mutans* is an oral bacterium, *B*. *subtilis* is mainly considered as a soil bacterium. However, *B*. *subtilis* was isolated from different areas associated with the dental environment^13,14^. Though, association of *B*. *subtilis* to dental diseases remains unclear^13,14^.

*S*. *mutans* is considered a primary causative bacterium of dental caries. *S*. *mutans’* virulence is due to its capability to form biofilms on the tooth surface^15^. Following initial adhesion, dietary sucrose is metabolized and transformed into glucans or fructans. These polysaccharides either serve as EP or remain associated with the cell as intracellular polysaccharides^15,16^.

*B*. *subtilis* is a Gram-positive non-pathogenic bacterium. When biofilm formation is triggered, *B*. *subtilis* cells stick together by producing an extracellular matrix^17^. The matrix primarily consists of EP synthesized by the products of the *epsA-O* operon, and ALF encoded by *tasA* located in the *tapA-sipW-tasA* operon^18^. The importance of TasA in a mono-species biofilm setting was described previously, both in cell-cell interactions^19^ and biofilm formation^20^. In the mono-species setting, TasA is required for development of complex colony architecture^21^ and provides a structure that can hold the cells together^22^. It is not yet known if TasA is responsible for similar interactions between different species of bacteria in a multi-species biofilm.

In the present study, *S*. *mutans* and *B*. *subtilis* were selected as model bacteria for investigating interactions of dual-species biofilm formation, and to investigate the interaction between the precursor for ALF -  TasA of *B*. *subtilis* and the dextran-associated EP of *S*. *mutans*. Dextran is a typical polymer found in biofilm extracellular matrix^23,24^, moreover, due to being a glucose homo-polymer with α-linkages, it is a relatively simple chemical moiety which may be used as a model for a larger group of glucans^23^. *B*. *subtilis* was chosen for its single gene encoding the TasA. Moreover, both bacteria can be found in the same habitat and therefore interact biologically, which indicated this model can appear in the natural environment. The aims of the current study were to determine the direct interaction between the model bacteria *B*. *subtilis* and *S*. *mutans* and to identify the role of TasA, a precursor for ALF produced by *B*. *subtilis*, in the formation of this co-species biofilm.

## Results

### *B. subtilis* and *S. mutans* form complex structures within dual-species biofilm

**Figure 1:**
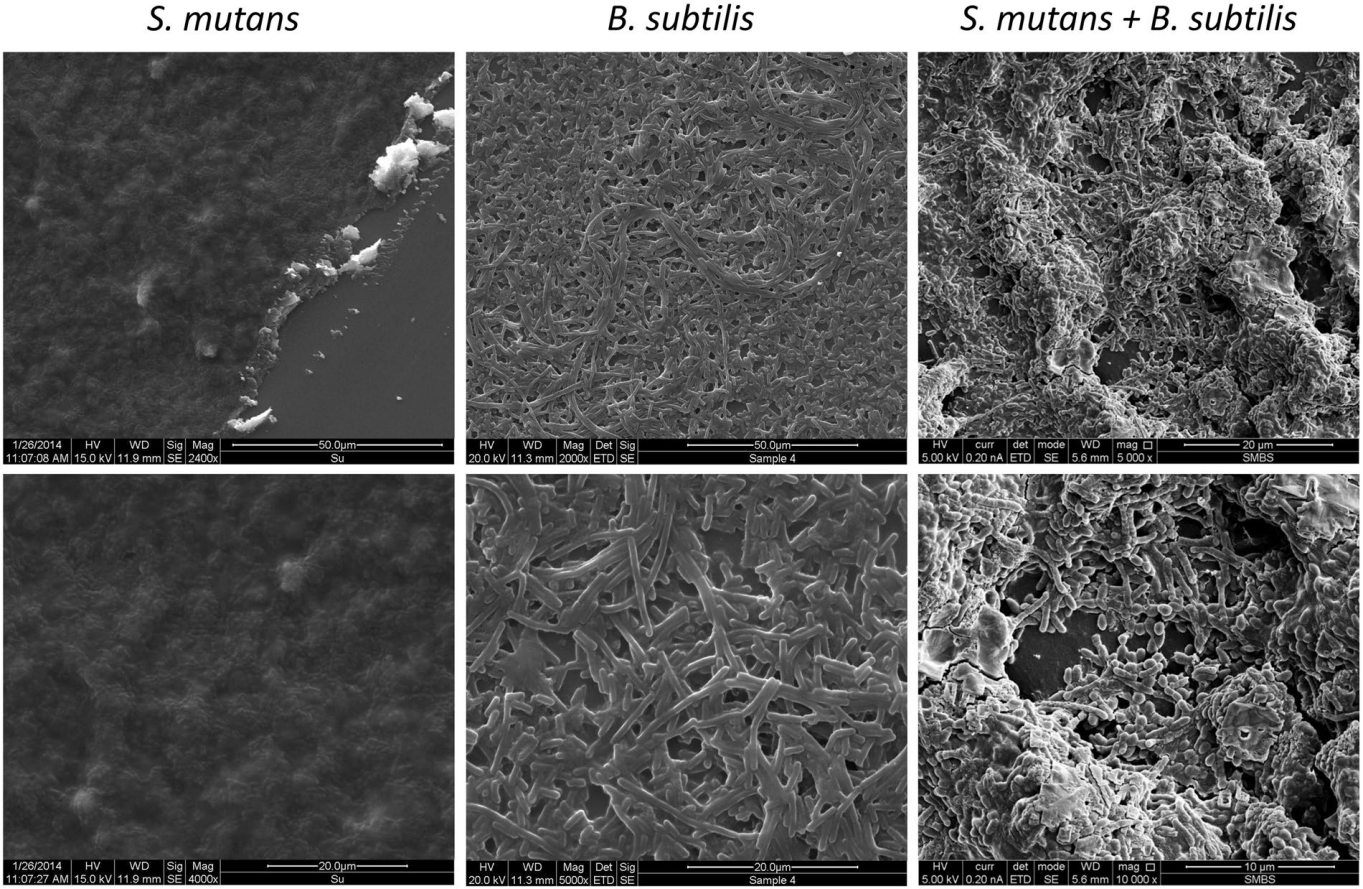
formation of complex structures during dual-species biofilm development. *S. mutans* and *B. subtilis* were grown in mono or dual-species biofilms. All biofilms were fixed with 4% Formaldehyde. The samples were taken for visualization under SEM. The lower panel is a higher magnification of the upper panel. The pictures represent the mono and dual-species biofilm. The complexity of the dual biofilm in comparison to the mono-species biofilm and the arrangement of both of the bacteria in the dual-species biofilm.

The first aim of this study was to determine whether an interaction between *B*. *subtilis* and *S*. *mutans* cells may result in formation of dual-species biofilm. We compared the morphology of mono and dual-species biofilms using scanning electron microscopy (SEM). *S*. *mutans* mono-species biofilm was homogenous and surrounded by EP. *B*. *subtilis* mono-species biofilm contained bundles of filamentous cells. The dual-species biofilm had more three-dimensionally complex structures than the mono-species biofilm (Fig. 1, right panel). Moreover, our observations showed close proximity between the rod shaped *B*. *subtilis* and the *S*. *mutans* cocci (Fig. 1).

### *B. subtilis* cell initially interacts with *S. mutans* biofilm via TasA

The co-localization of *B*. *subtilis* and *S*. *mutans* in the dual-species biofilm indicated a direct interaction between these bacterial cells. To explore this interaction, we quantified adhesion forces between the two species by single cell force spectroscopy (SCFS) using an atomic force microscope (AFM). We quantified adhesion forces between single *B*. *subtilis* cells and a glass coverslip surface (control sample), a *B*. *subtilis* biofilm and a *S*. *mutans* biofilm.

**Figure 2:**
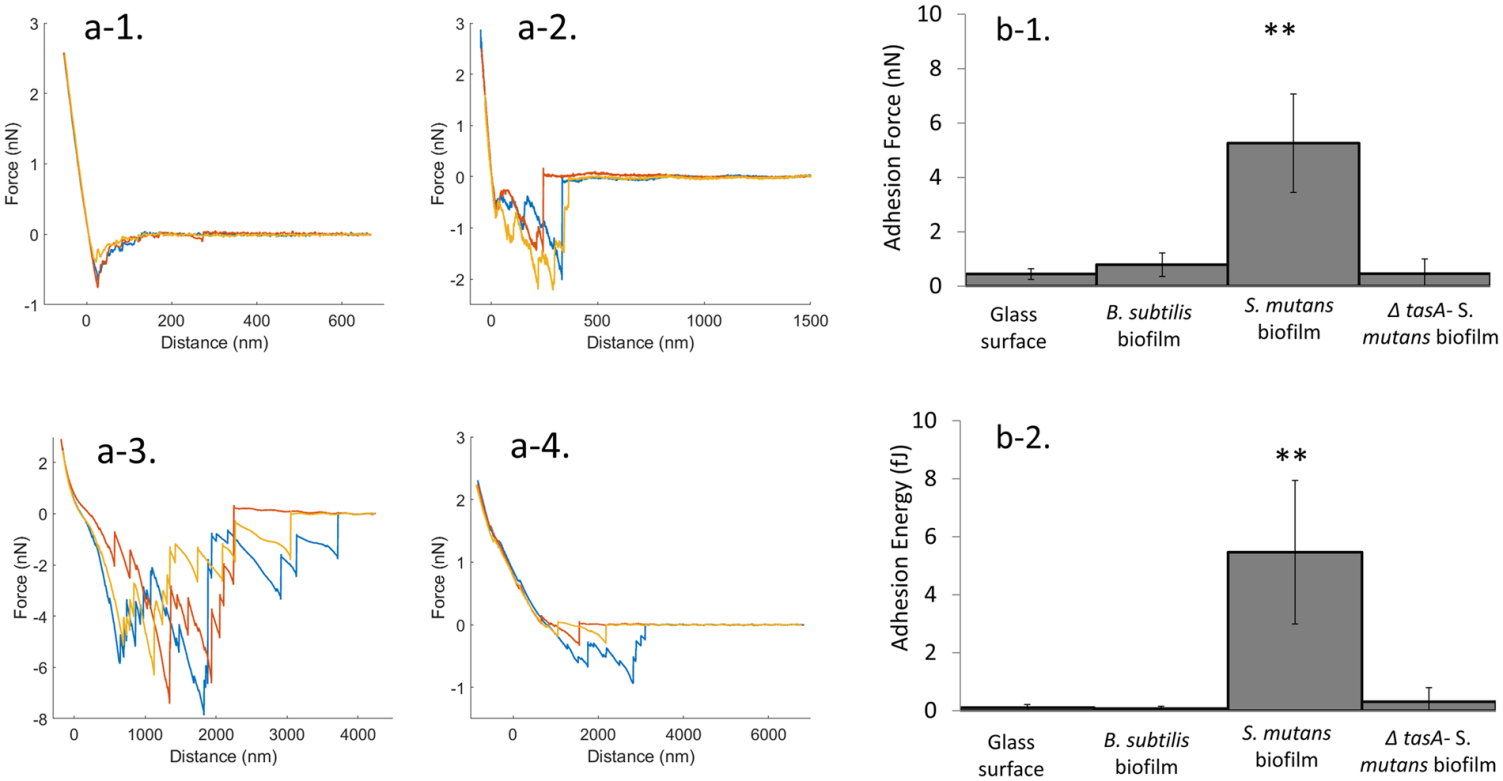
*B. subtilis* cell interacts with *S. mutans* biofilm via TasA protein. *B. subtilis* bacterial cells were incubated on coverslip for 20 minutes until attachment to the glass. One *B. subtilis* cell was picked and attached to tipless cantilever coated with PDA. Single cell force spectroscopy measurements were taken using AFM. (**a**) representative force profiles of *B. subtilis* WT on a glass surface (a-1), *B. subtilis* WT on a *B. subtilis* biofilm (a-2), *B. subtilis* WT on *S. mutans* biofilm (a-3) and *B. subtilis ∆tasA* on *S. muatns* biofilm (a-4). (**b**) Adhesion force (b-1) and energy (b-2) of single *B. subtilis* cell to glass, *B. subtilis* biofilm and *S. mutans* biofilm. The adhesion force and energy between *B. subtilis* cell and *S. mutans* biofilm was significantly higher compare to glass. However, *∆tasA* strain did not show a high adhesion force or energy towards *S. mutans* biofilm. The data are displayed as a mean value ± standard deviation. **P value < 0.01 compared to control.

The first step was the attachment of a single *B*. *subtilis* cell onto a tipless cantilever coated with polydopamine (PDA) (Supplementary Fig. S1). Next, the cantilever was repeatedly approached to and retracted from the test surface to determine the adhesion energy, the maximum adhesion force, and the distance at which the bond ruptures as the cantilever is retracted from the surface (Fig. 2 a1-4). While the force profile of *B*. *subtilis* on glass featured weak interactions at short distances, the force profile between a single *B*. *subtilis* cell and *B*. *subtilis* biofilm showed multiple rupture events at longer distances. The force profiles of *B*. *subtilis* cells and *S*. *mutans* biofilm showed increasing number of rupture events, higher adhesion force, and longer rupture length, suggesting interaction with multiple adhesins that could be stretched from the cell surface. The strong interaction between *B*. *subtilis* and *S*. *mutans* was also reflected in the number of adhesion events observed in force spectroscopy analysis. *B*. *subtilis* adhered to *S*. *mutans* biofilm in 100% of the measured force curves, while up to 40% of force curves showed no adhesion when *B*. *subtilis* was approached to a glass surface or a *B*. *subtilis* biofilm.

Adhesion is described by the maximum adhesion force measured between the bacterium and the sample (Fig. 2b-1), and the adhesion energy calculated from the area under the force-distance curve (Fig. 2b-2). The adhesion force between the *B*. *subtilis* cell and the *B*. *subtilis* biofilm was 2-fold higher than the adhesion force to glass. However, its adhesion force to *S*. *mutans* biofilm was 5-fold higher (Fig. 2b). The adhesion force between *B*. *subtilis* cells and *S*. *mutans* biofilm was so high that it frequently caused detachment of *B*. *subtilis* from the cantilever.

**Figure 3:**
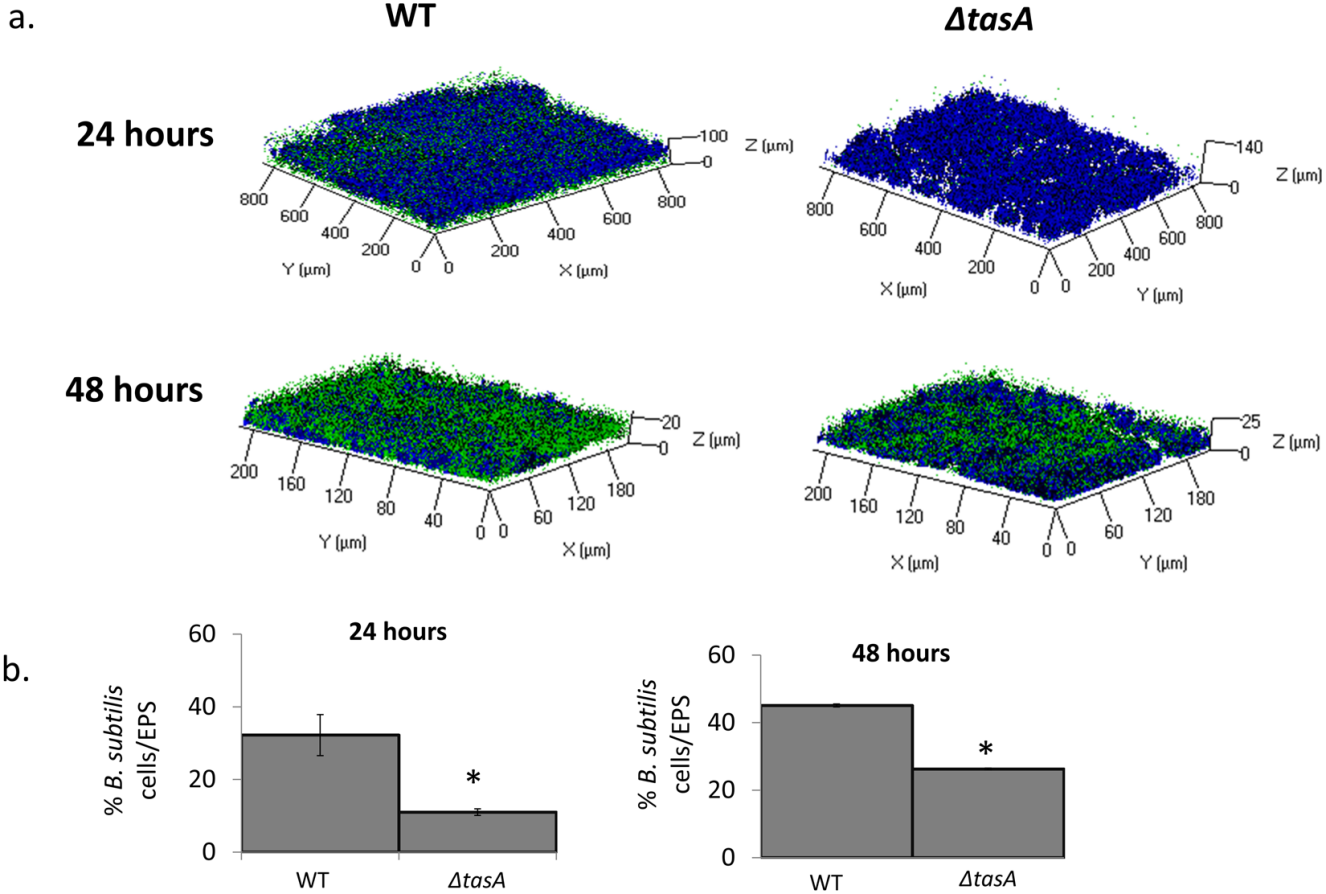
*B. subtilis’* TasA protein effects the structure of co-species biofilm and the incorporation of *B. subtilis* into co-species biofilm. (**a**) Representing CLSM picture of co-species biofilm. *B. subtilis* (WT (YC161 marked with GFP) or *ΔtasA* (marked with GFP) and *S. mutans* were grown together for 24 h (upper panel) or 48 h (lower panel). *S. mutans* dextran-associated EP were marked in blue using Alexa fluor 647. In both of the time periods, there are more WT *B. subtilis* cells among the *S. mutans’* dextran-associated EP compare to the *ΔtasA* strain. Moreover, after 48 h there is an increase in the amount of incorporated *B*. *subtilis* cells within the dual-species biofilm in both strains (WT and *ΔtasA*). (**b**) Quantification of the fluorescent intensity of GFP for live bacteria and Alexa fluor for EP. The data are displayed as a mean value of data from five biological repeats each performed as triplicate. The amount of WT *B. subtilis* cells is significantly higher than the *ΔtasA* strain after 24 and 48 h. Furthermore, there is an increase in the amount of *B. subtilis* cells within the dual-species biofilm in both of the strains. The data are displayed as a mean value ± standard deviation. *P value < 0.05 compared to WT.

**Figure 4:**
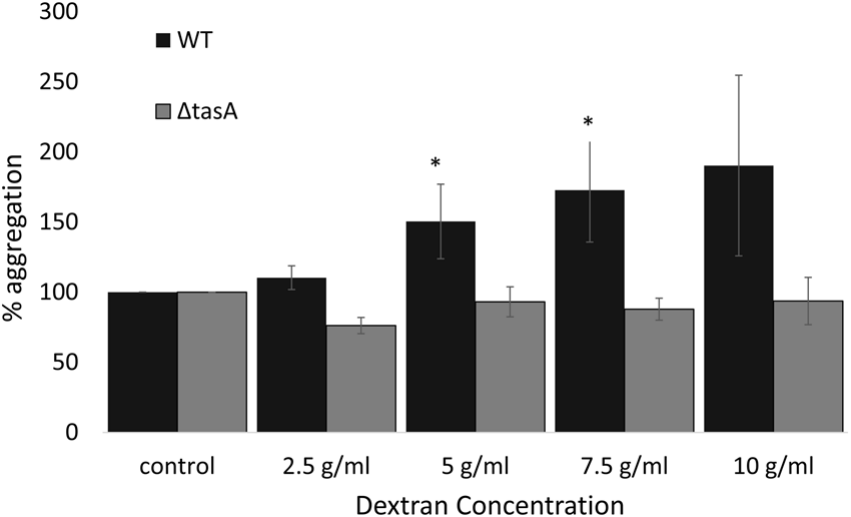
TasA protein is essential for dextran-induced *B. subtilis* aggregation. *B. subtilis* cells were centrifuged and washed twice using aggregation buffer. The bacterial cells were incubated in aggregation buffer supplemented with different concentrations of dextran. O.D (600 nm) measurements were taken every 30 min. Final aggregation percentages were calculated. The data are displayed as a mean value ± standard deviation of data from three biological repeats each performed as duplicates. ANOVA following post-hoc t-test with Bonferroni correction has been conducted. *P value < 0.05 compared to control.

It has been established that TasA is a cell wall associated protein that plays a crucial role in *B*. *subtilis’* biofilm formation^20^. Therefore, the next step was to examine the possible role of TasA in interaction between *B*. *subtilis* and other bacteria in mixed-species biofilm. Using SCFS, we were able to measure the initial adhesion force of Δ*tasA* mutant strain of *B*. *subtilis* to *S*. *mutans* biofilm. The force profiles obtained for the Δ*tasA* mutant towards glass (data nor shown) or *S*. *mutans* biofilm were similar to those of the WT strain (Fig. 2a-4). However, in contrast to the WT *B*. *subtilis*, the Δ*tasA* mutant showed weak interaction with *S*. *mutans* biofilm (Fig. 2b).

### TasA affects the structure and the incorporation of *B. subtilis* into co-species biofilm

Next, confocal laser scanning microscopy (CLSM) was used to observe the three-dimensional structure of dual-species biofilm. In Fig. 3, dextran-associated EP, formed by *S*. *mutans*, is labelled with dextran alexa fluor (blue), and *B*. *subtilis* cells are tagged with GFP (green). The CLSM images indicate that *B*. *subtilis* cells were not only attached to the upper layer of the biofilm but also appear in deeper layers and are surrounded by dextran-associated EP (Fig. 3a). However, *B*. *subtilis* Δ*tasA* cells (green) were less abundant in the dual-species biofilm (Fig. 3b) after 24 hours. *B*. *subtilis* (both in WT and *ΔtasA*) increased in abundance after 48 hours, but Δ*tasA* remained less abundant than the WT *B*. *subtilis* (Fig. 3). These results indicate that TasA contributes to establishment of *B*. *subtilis* in co-species biofilm with *S*. *mutans*, especially in the initial stages.

### TasA is essential for dextran-induced *B. subtilis* aggregation

*S*. *mutans’* EP serve as a platform for bacterial adhesion^25^. Therefore, we tested whether dextran (a primary component in *S*. *mutans’* EP^23,26^) contributes to the attachment of *B*. *subtilis* cells. An aggregation assay showed that addition of increasing concentrations of dextran stimulated aggregate formation by WT cells of *B*. *subtilis* by 40–80%, while dextran supplementation could not notably affect the *ΔtasA* mutant cells (Fig. 4). The strains had similar aggregation ratio in the absence of dextran (data not shown).

### The amount of dextran-associated EP affects incorporation of *B. subtilis* cells to the dual-species biofilm

**Figure 5:**
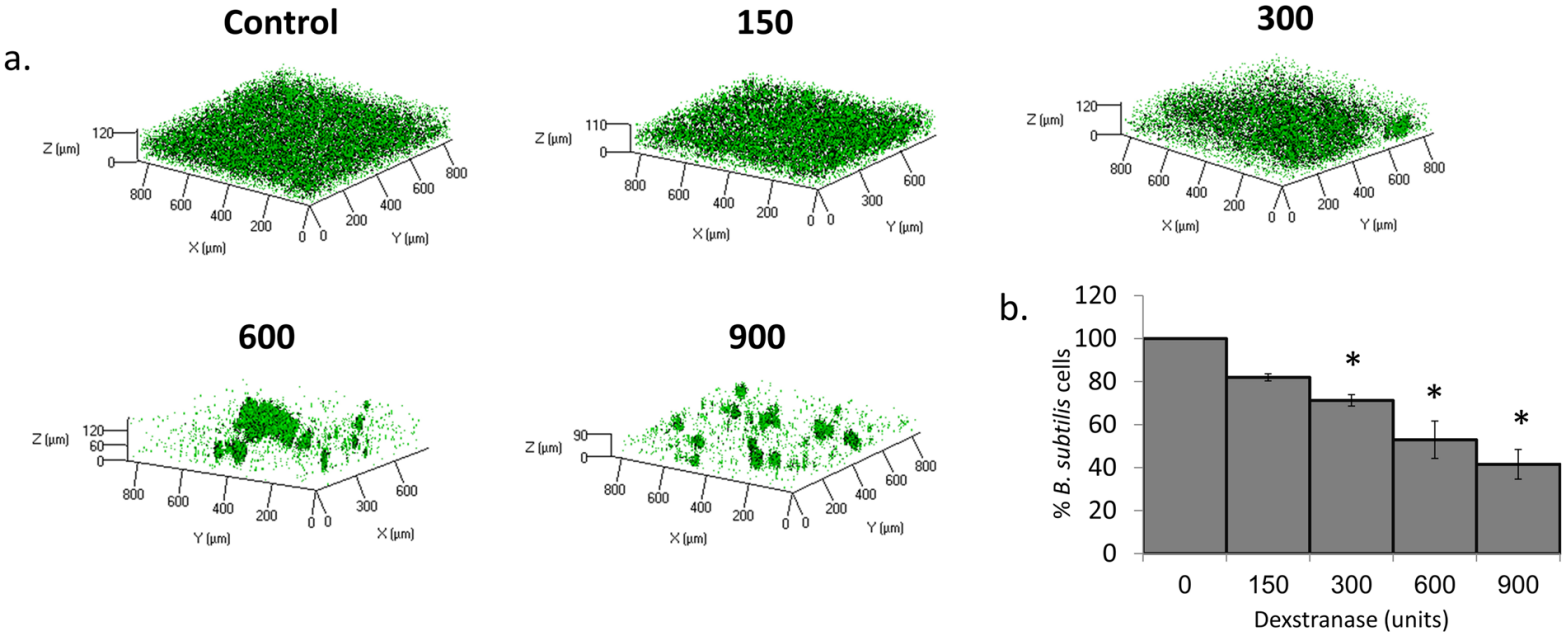
*B. subtilis* incorporation in the dual-species biofilm is highly dependent on the amount of dextran-associated EP. (**a**) Representing CLSM picture of co-species biofilm. *B. subtilis* (YC161 marked with GFP) and *S. mutans* were grown together for 24 h in the presence of different amounts of dextranase. As the amount of dextranases increased the amount of *B. subtilis* cells in the co-species biofilm decreased. (**b**) Quantification of the fluorescent intensity of GFP for *B. subtilis* cells. The data are displayed as a mean value of data ± standard deviation from two biological repeats each performed as triplicate. The amount of *B. subtilis* cells in the co-species biofilm significantly decreased in dose dependent manners with the increase in the amount of dextranase. *P value < 0.05 compared to control.

To investigate whether dextran affects *B*. *subtilis* biofilm formation, we determined if enzymatic removal of dextran during biofilm formation affected the abundance of *B*. *subtilis*. We visualised *B*. *subtilis* WT cells in dual-species biofilm formed in the presence of different dextranase concentrations. The abundance of *B*. *subtilis* biomass decreased in a dose-dependent manner in the dual-species biofilm in response to increasing concentration of dextranase (Fig. 5), suggesting that the quantity of dextran is important for *B*. *subtilis* adherence in dual-species biofilm.

### Expression of *tapA* operon of *B. subtilis* is up-regulated in the presence of *S. mutans*

**Figure 6:**
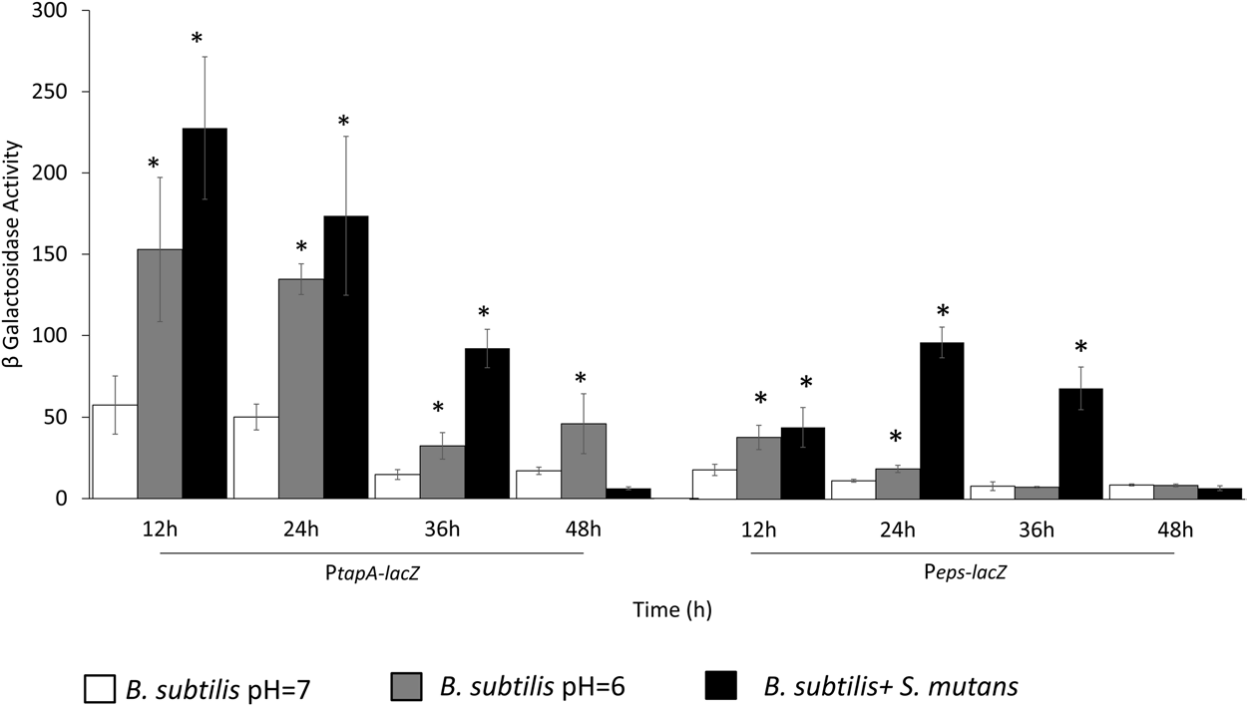
Expression of the *tapA* and *eps* operon in *B. subtilis* are up regulated in the presence of *S. mutans*. *B. subtilis* (P_*tapA*-*lacZ*_ or P_*eps*-*lacZ*_) cells were grown in the presence or absence of *S. mutans* cells either in pH7 or in pH6. Samples were collected every 12 hours for β galactosidaze assay. The data are displayed as a mean value ± standard deviation of data from three biological repeats each performed as triplicates. The expression of the *tasA* and the *eps* operons was significantly induced in the presence of *S. mutans*. The expression time of both of the operons was not in parallel. While *tasA* expression was significantly increased after the first 12 h of incubation, the higher induction in *eps* expression was after 36 h. *P value < 0.05 compared to *B. subtilis* pH = 7.

Finally, we aimed to determine whether the presence of *S*. *mutans* affects expression of the *tapA-sipW-tasA* operon. The β galactosidase assay showed approximately 5-fold up-regulation in the activity of the *tapA* promoter during the first 12 h of co-culture, although the activity decreased over time. *S*. *mutans* is a very acidogenic bacterium which produces a high amount of acid during its growth. We therefore tested, if its effect on gene expression was through modifying the pH in the media. A shift in the pH from 7 to 6 up-regulated the *tapA* promoter activity, although to a lesser extent than the up-regulation observed in the presence of *S*. *mutans* (Fig. 6a).

Since *ΔtasA* mutant appeared in the dual-species biofilm after 24 hours and furthermore, the increase in the amount of the mutant cells after 48 hours indicated that another factor may be involved in the attachment of *B*. *subtilis* to the dual-species biofilm. Therefore, the next step was to determine whether the presence of *S*. *mutans* affects the expression of the *epsA-O* operon. The β galactosidase assay showed approximately 2.5-fold up-regulation in the expression of the *eps* promoter during the first 12 h of co-culture. In contrast to the *tapA* operon, the highest up-regulation in the *eps* operon was only after 24 hours. These results indicate that the regulation of both of operons is not stimulated simultaneously in the dual-species biofilm.

## Discussion

The current study aimed to explore the role of TasA in the interaction between *B*. *subtilis* and *S*. *mutans* in dual-species biofilm. Our results point to an important role for interaction between two matrix components - *B*. *subtilis’* TasA protein and *S*. *mutans’* dextran polysaccharides - during dual-species biofilm formation.

Biofilm formation begins with attachment of bacteria to the substrate. Attachment involves initial reversible adhesion, which occurs within seconds to minutes, followed by irreversible adhesion, which results in bacterial aggregation^27,28^. The high sensitivity and resolution of the AFM allows quantification of adhesion forces of a single bacterial cells to probe the interaction forces that govern initial attachment. Therefore, the initial reversible interaction of *B*. *subtilis* and *S*. *mutans* cells was determined using SCFS. The low adhesion force between *B*. *subtilis* and glass explains the inability of *B*. *subtilis* NCIB3610 to adhere to surfaces and to form submerged biofilm at solid-liquid interfaces^29^. *B*. *subtilis* adhered more strongly to *B*. *subtilis* biofilm, but its adhesion force to *S*. *mutans* biofilm was significantly stronger. The relatively high inter-species adhesion force explains the capability of *B*. *subtilis* to form submerged biofilm in cooperation with *S*. *mutans*, which more easily colonizes the glass substrate.

The importance of TasA in biofilm formation by *B*. *subtilis* is well established in the literature^20,22^. Additionally, the role of ALF in cell-host adhesion was demonstrated in other bacterial species such as *Escherichia coli* and *Salmonella enteritidis*^30,31^. We show here that TasA was critical for the interaction between *B*. *subtilis* cells to *S*. *mutans* biofilm, as a TasA deficient mutant adhered only weakly to *S*. *mutans* (Fig. 2) and was less abundant in the dual-species biofilms (Fig. 3).

The role of TasA in the initial formation of dual-species biofilm raised the question of whether the presence of *S*. *mutans* affected the expression of *tasA*. Expression of the *tapA-sipW-tasA* operon increased in the presence of *S*. *mutans* during the first 12 h of co-culture, and subsequently decreased after 36 h, implying that TasA is required in early stages of dual-species biofilm development. Bacteria are known to differentiate into biofilm-forming cells in response to environmental cues^32,33^. Thus, induction in the expression of *tapA-sipW-tasA* operon may be triggered by the production of an organic acid(s) by *S*. *mutans* and a decrease in pH levels due to sucrose metabolism^34,35^. Therefore, *tapA-sipW-tasA* expression was examined in a medium with decreased pH by addition of lactic acid, a major end-product of glycolysis by *S*. *mutans*^36^. The *tapA* expression was up-regulated at pH 6, indicating a pH-dependent induction of the *tapA* operon. These results support the findings by Chen *et al*.^37^, who found that organic acids stimulate biofilm formation by *B*. *subtilis*^37^.

We hypothesized that TasA interacted with a major matrix component produced by *S*. *mutans*, namely dextran. Dextran induced aggregation by *B*. *subtilis* cells in a dose dependent manner, and the amount of dextran affected the abundance of *B*. *subtilis* cells in the dual-species biofilm. These results are in accordance with previous studies that showed the importance of *S*. *mutans* EP in accumulation and adherence of other bacterial species to the biofilm^25,38,39^. Interestingly, the effect of dextran on *B*. *subtilis* aggregation was absent in the *ΔtasA* mutant suggesting that the interaction between TasA and *S*. *mutans* biofilm is due to ALF-EP interaction. This result is supported by Romero *et al*.^22^, who suggest that TasA ALF interact in a specific way with EP matrix^22^ and Besingi *et al*.^40^ who suggested that amyloid proteins can be adhesive to glucans^40^. The aggregation of *B*. *subtilis* in the presence of dextran demonstrates the irreversible interactions that occur between the bacteria. Our results support the finding of Diehl *et al*.^41^ who suggested, based on nuclear magnetic resonance (NMR) spectra, that TasA interacts specifically with EP. Such interactions can explain the strong adhesion of *B*. *subtilis* cells to *S*. *mutans* biofilm, as well as the reduced adherence of the *∆tasA* strain. Moreover, their study showed that EP promotes the restructuring of TasA into ALF, which may explain the increased aggregation of *B*. *subtilis* WT in the presence of Dextran^41^.

A recent study by Besingi *et al*.^40^ found amyloid fibers in *S*. *mutans* biofilms. However, we did not detect any ALF in the *S*. *mutans* biofilms. This could be due to the use of sucrose in the growth media, as glucans formed from sucrose can mask amyloid production^40^. However, the existence of such fibers may also contribute to the adhesion of more bacterial species and the complexity of the formed biofilm in multispecies settings.

Overall, this study demonstrates that the interaction between TasA produced by *B*. *subtilis* and EP produced by *S*. *mutans* plays an essential role in initial attachment of bacteria during dual-species biofilm formation. Moreover, we demonstrate the complexity of how each matrix component can influence multispecies biofilm formation. This study provides new insights into ALF contribution to the structural complexity of dual-species biofilm. A clear view of this interaction may result in new approaches to control biofilm formation and adhesion of these specific bacterial species. Moreover, further investigation of the specific active sites on such extracellular matrix components may lead to developing targeted treatments to combat biofilms.

## Materials and Methods

### Strains and growth media

The cultures of clinical isolate strain *S*. *mutans* UA159 were grown overnight in brain heart infusion broth (BHI, Acumedia, Landing, Michigan) at 37 °C in 95% air/5% CO_2_. The following *B*. *subtilis* strains were used in this study: NCIB3610 (WT), YC161 (*P*_*spank*_*-gfp*), DDA (*∆tasA, P*_*spank*_*-gfp*), RL4582 (*P*_*tapA*_*-lacZ*) and BS505 (*∆tasA*). For starter culture generation, one colony of *B*. *subtilis* from fresh Lysogeny broth (LB, Neogen, Lansing, Michigan, USA) agar plate was grown in LB and incubated at 37 °C at 150 rpm for 5 h. For all the experiments, both bacteria species were collected in late exponential phase.

### Mono and dual-species biofilm formation

For *B*. *subtilis* mono-species biofilm, an overnight culture (30 °C at 150 rpm) was diluted 1:10 (to obtain final O.D (600 nm) = 0.07) into LB containing 3% lactose, and incubated at 37 °C at 50 rpm for 5 h^42^. For *S*. *mutans* mono-species biofilm, in 96 well plate (Nunc, Roskilde, Denmark), overnight cultured *S*. *mutans* (O.D (600 nm) = 1) were diluted 1:10 into fresh BHI supplemented with 2% sucrose. The plate was incubated at 37 °C in 95% air/5% CO_2_ for 24 or 48 h. The medium was replaced with fresh medium after 24 h of incubation^34^. For dual-species biofilm, cells of *B*. *subtilis* and *S*. *mutans* were grown in 96 well plate as follows: to fresh BHI supplemented with 2% sucrose were introduced the overnight cultured *S*. *mutans* (diluted by a ratio 1:10, approximately 2.5 × 10^7^ CFU) and *B*. *subtilis* (diluted by a ratio 1:100, approximately 2.5 × 10^5^ CFU). The ratio between *B*. *subtilis* and *S*. *mutans* cells that were seeded to obtain the dual-species biofilm was approximately 1:100. The plate was incubated at 37 °C in 95% air/5% CO_2_ for 24 or 48 h. The medium was replaced with fresh medium after 24 h.

### Visualization of mono and dual-species biofilms by SEM

SEM analysis was used for initial visualization of the generated biofilms mainly in terms of structure and the spatial organizations in multispecies biofilm. For *B*. *subtilis* mono-species biofilm: a sample of 10 µl from the culture containing the *B*. *subtilis* mono-species biofilm was placed on small cover slip^43,44^. The *S*. *mutans* mono and dual-species biofilms were grown as described above on sterile coverslip (5 mm diameter, glass, Barnaor, Israel). The coverslips were removed and washed twice with sterile demineralized water (DDW). All samples were fixed using 4% formaldehyde for 20 min and washed using DDW. The samples were visualized using an analytical Quanta 200 Environmental High Resolution Scanning Electron Microscope (EHRSEM) (FEI, Eindhoven, The Netherlands).

### Single cell force spectroscopy

SCFS was used to determine the initial interaction between *B*. *subtilis* cell and *S*. *mutans* biofilm. The experiment was conducted as described by Zeng *et al*.^45^, previously with slight modifications^45^.

#### Probes preparation

Tipless cantilevers (CSC38/No Al/tipless, Mikromasch, Sofia, Bulgaria) with a length of 350 µm, width of 32.5 µm, a thickness of 1 µm a nominal spring constant of 0.03 N/m were incubated for 20 min under UV. Then the cantilevers were immediately transferred to a solution of 4 mg/ml dopamine (Sigma Aldrich, St. Louise, Missouri, USA) in 20 mM Tris buffer (pH 8.5) (Eastman Kodak company, Rochester, NY, USA), and incubated for 30 min before rinsing in triple distilled water.

#### *B. subtilis* cell attachment

Starter cultures of *B*. *subtilis* cells (WT or *ΔtasA*) were centrifuged at 3000 × *g* for 3 min and washed with phosphate buffer saline (PBS) three times. 75 µl of re-suspended cells were placed on a coverslip and incubated for 20 min at RT to allow attachment. The unattached bacteria were removed by washing with PBS. A tipless polydopamine-coated cantilever was approached to one attached bacteria with 5 nN force for 300 s, resulting in immobilization of the cell on the cantilever.

#### Biofilm preparation

*S*. *mutans* biofilm was prepared as described above. The coverslip was removed, washed twice with PBS and glued to glass-slide using epoxy (JPK, Germany).

#### AFM force measurements

Force measurements were conducted in PBS using JPK Nanowizard 3 (JPK instruments, Germany). The sensitivity of tipless cantilevers was calibrated on a glass surface in PBS and the spring constant was calibrated using the thermal fluctuation method (included in the AFM software). After immobilization of a bacterial cell on the cantilever, force spectroscopy was performed by approaching the cantilever with the immobilized bacterium to either bare glass or biofilms of *B*. *subtilis/S*. *mutans*, to determine the maximum adhesion force of the cell to each sample. Force curves were measured with a maximum force of 2.5 nN and 15 s extend delay and repeated 10 times at three different positions. Measurements with each bacterium were conducted for a period of approximately 20 min. To ensure that the bacterium was still attached to the cantilever and that it retained its position, optical microscopy was used during the measurements when possible (glass, and *B*. *subtilis* biofilm). For measurements on *S*. *mutans* biofilm, visualization of the bacteria on the probes was conducted before and after each measurement and when a change in the force profile was observed. The images were taken using The images were taken using a x40 objective lens, Ti-E microscope (Nikon Instruments, Melville, NY, USA) and a optiMOS^TM^ sCMOS Camera (Qimagimg, Surrey, Canada) (Sup Fig. 1). Each measurement was performed using at least two different bacterial cells and/or biofilm samples. In detail: *B*. *subtilis* WT on a glass: 3 bacteria, 117 force curves, adhesion frequency: 73.5% *B*. *subtilis* WT on a *B*. *subtilis* biofilm: 2 bacteria on 2 different biofilms, 118 force curves adhesion frequency: 85.6%, *B*. *subtilis* WT on *S*. *mutans* biofilm: 3 bacteria on 2 different biofilms, 53 force curves adhesion frequency: 100%, *B*. *subtilis ∆tasA* on glass: 3 bacteria, 46 force curves adhesion frequency: 61%, *B*. *subtilis ∆tasA* on *S*. *mutans* biofilm: 2 bacteria on one biofilm 100 force curves adhesion frequency: 51%.

Importantly, control measurements between a PDA coated cantilever and, glass, *B*. *subtilis* biofilm and *S*. *mutans* biofilm were taken (Supplementary Fig. S2).

#### AFM measurements Analysis

Analysis was conducted using JPK data processing software. The adhesion force was considered as the highest attraction force in the curve (largest negative force), and the detachment work energy was evaluated by calculating the area between the curve and the baseline. Curves not exhibiting adhesion events were discarded from the adhesion force and energy calculation.

### Characterization of biofilm structure by CLSM

CLSM was used for visualization of deeper layers of the biofilm and quantification of *B*. *subtilis* cells in the dual-species biofilm. Dual-species biofilm was prepared as described above using *B*. *subtilis* cells tagged with GFP. Incorporation of dextran-associated EP was visualized by adding 1 µM Alexa Fluor 647-labeled dextran conjugate (molecular weight, 10,000 MW, Molecular Probes Inc.) to the growth media, during biofilm formation as described previously^38^.

The generated biofilms were washed carefully using PBS and visualized by Zeiss LSM510 CLS microscope (Carl Zeiss, Oberkochen, Germany). Three-dimensional images were constructed using Zen software (Carl Zeiss). At least three random fields were observed in five independent experiments. The amount of EP produced by *S*. *mutans* and viable *B*. *subtilis* cells was calculated as blue and green fluorescence intensity, respectively, using ImageJ software (http://rsb.info.nih.gov/ij).

### *B. subtilis* aggregation in the presence of dextran

The effect of dextran, the primary polysaccharide in *S*. *mutans’* EP^23,26^, on *B*. *subtilis* aggregation was evaluated using an aggregation assay. Starter cultures of *B*. *subtilis* cells (WT or *ΔtasA*) were grown as described above. The cells were centrifuged at 3000xg for 10 min and washed twice in aggregation buffer (1 M potassium buffer pH = 7.5). The bacterial cells were re-suspended in aggregation buffer supplemented with varying concentrations of dextran (MW = 11,800 g/mol) to resemble *S*. *mutans* EP. O.D (600 nm) was measured every 30 min for 2 h. Aggregation percentage was calculated by^46^:

$$\frac{O{D}_{initial}-O{D}_{finall}}{O{D}_{initial}}\times 100.$$

### Dextran effect on *B. subtilis* adhesion

The effect of dextran on the incorporation of *B*. *subtilis* to the dual-species biofilm was tested using increased concentrations of dextranase^47,48^. Dextranase solution was prepared by dissolving 3 mg of lyophilized dextranase enzyme (Sigma Aldrich, St. Louise, Missouri, USA) in 1 ml of PBS. The dextranase solution was added to the dual-species formed biofilms in different amounts equal to: 156, 300, 600 and 900 units of enzyme. The generated biofilms were taken for visualization by CLSM as mentioned above. The amount of *B*. *subtilis* cells were calculated by fluorescent intensity using ImageJ software.

### Quantification of *tasA* and *eps* operons expression

To analyse the effect of *S*. *mutans* presence on *tasA* and *eps* expression, we used *B*. *subtilis* P_*tapA*-*lacZ*_ or P_*eps*-*lacZ*_ strains. *B*. *subtilis* cells were grown separately or with *S*. *mutans*, in BHI supplemented with 2% sucrose. To determine the effect ∆pH on *tasA* expression, 30% lactic acid solution (Merck & Co., Inc. Kenilworth, New Jersey, US) was used for the adjustment of pH of the medium to pH 6. Samples were collected every 12 h*;* normalization conducted using CFU on selective plates (streptomycin-LB agar plates). O.D was calculated according to 1 O.D (600 nm) = 1.5*10^8 CFU^49^. β-Galactosidase activity assay was conducted as described previously^50^.

### Statistical analysis

The obtained data were statistically analysed using t-test. When needed ANOVA following post-hoc t-test with Bonferroni correction was conducted. All tests applied were two-tailed, and a p-value of 5% or less was considered statistically significant. Statistical analysis was performed using Microsoft Excel software.

### Data availability

The datasets generated and analysed during the current study are available from the corresponding author on reasonable request.

## Electronic supplementary material

Supplementary Dataset 1
