# Supplementary material for: Cell wall associated protein TasA provides an initial binding component to extracellular polysaccharides in dual-species biofilm
Source: Sci Rep. 2018 Jun 19;8:9350. doi: 10.1038/s41598-018-27548-1 (PMC6008451; doi:10.1038/s41598-018-27548-1)
Supplement: Supplementary file 1 — Supplementary Dataset 1 [file 41598_2018_27548_MOESM1_ESM.docx]

**Supplementary Material**

Cell wall associated protein TasA provides an initial binding component to extracellular polysaccharides in dual-species biofilm

Danielle Duanis-Assaf ^a,b^, Tal Duanis-Assaf ^c^, Guanghong Zeng ^d^, Rikke Louise Meyer ^d^, Meital Reches ^c^, Doron Steinberg ^b^ and Moshe Shemesh ^a*^

^a^Department of Food Quality and Safety, Institute for Postharvest Technology and Food Sciences, Agricultural Research Organization (ARO), Volcani Center.

^b^Biofilm Research Laboratory, Institute of Dental Sciences, Faculty of Dental Medicine, Hebrew University-Hadassah.

^c^Institute of Chemistry, Hebrew University of Jerusalem, Jerusalem, Israel.

^d^iNANO, Aarhus University, Aarhus C, Denmark.

^*^Correspondence should be addressed to: Moshe Shemesh, Department of Food Quality and Safety, Institute of Postharvest Technology and Food Sciences, ARO the Volcani Center, Derech Hamacabim, POB 15159, Rishon LeZion 7528809, Israel; Email: [moshesh@agri.gov.il](mailto:moshesh@agri.gov.il).

**Content**

Supplemental Figures:

Fig. S1, Fig. S2


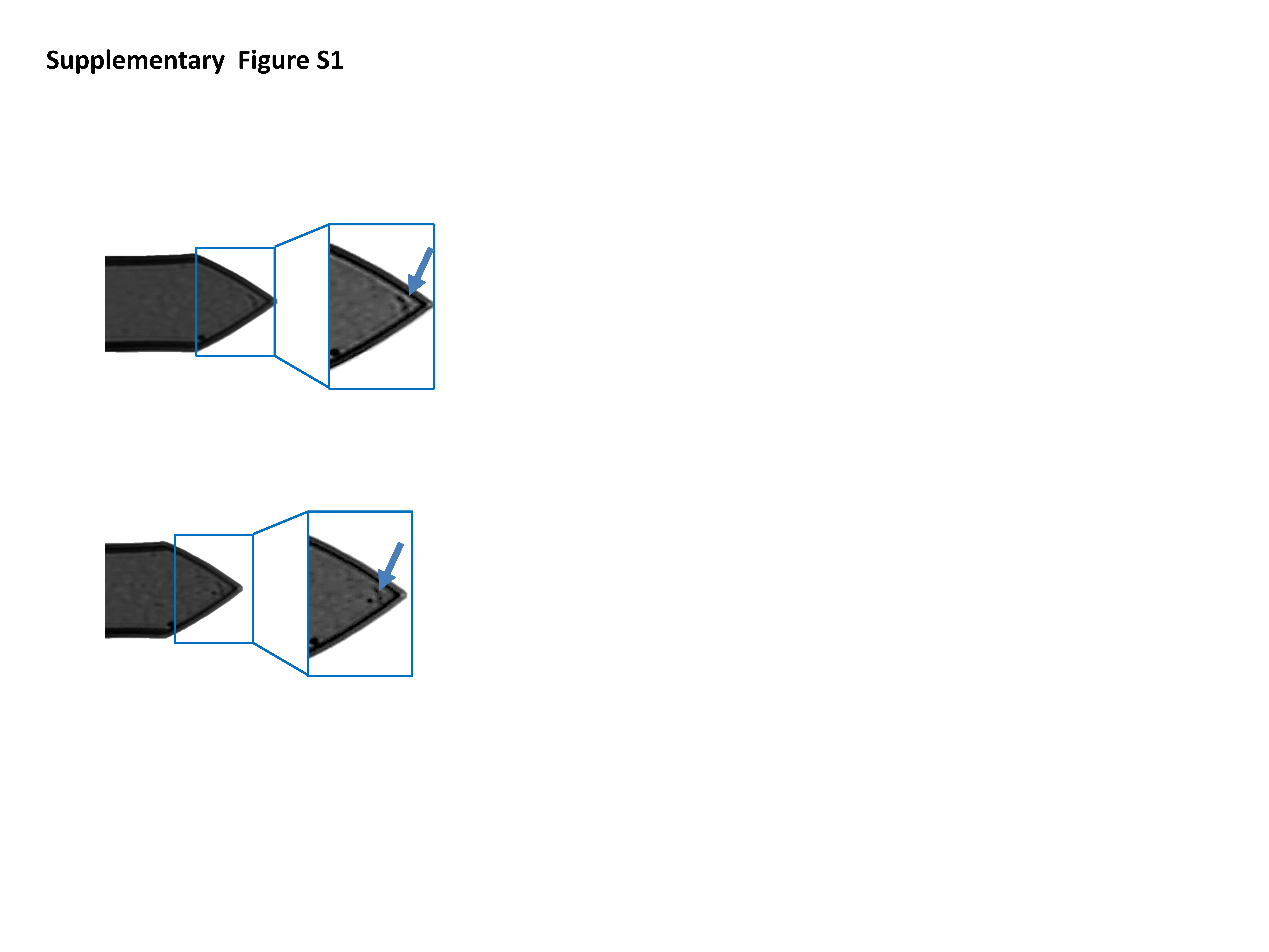


Supplementary Figure S1. representative images of a *B. subtilis* cell attached to AFM cantilever before and after measurements.

The top panel is a *B. subtilis* cell attached to tipless cantilever coated with PDA before AFM measurements and the lower panel is the same bacterium after finishing the AFM measurements. The images were taken using a x40 objective lens, Ti-E microscope (Nikon Instruments, Melville, NY, USA) and a optiMOS^TM^ sCMOS Camera (Qimagimg, Surrey, Canada)


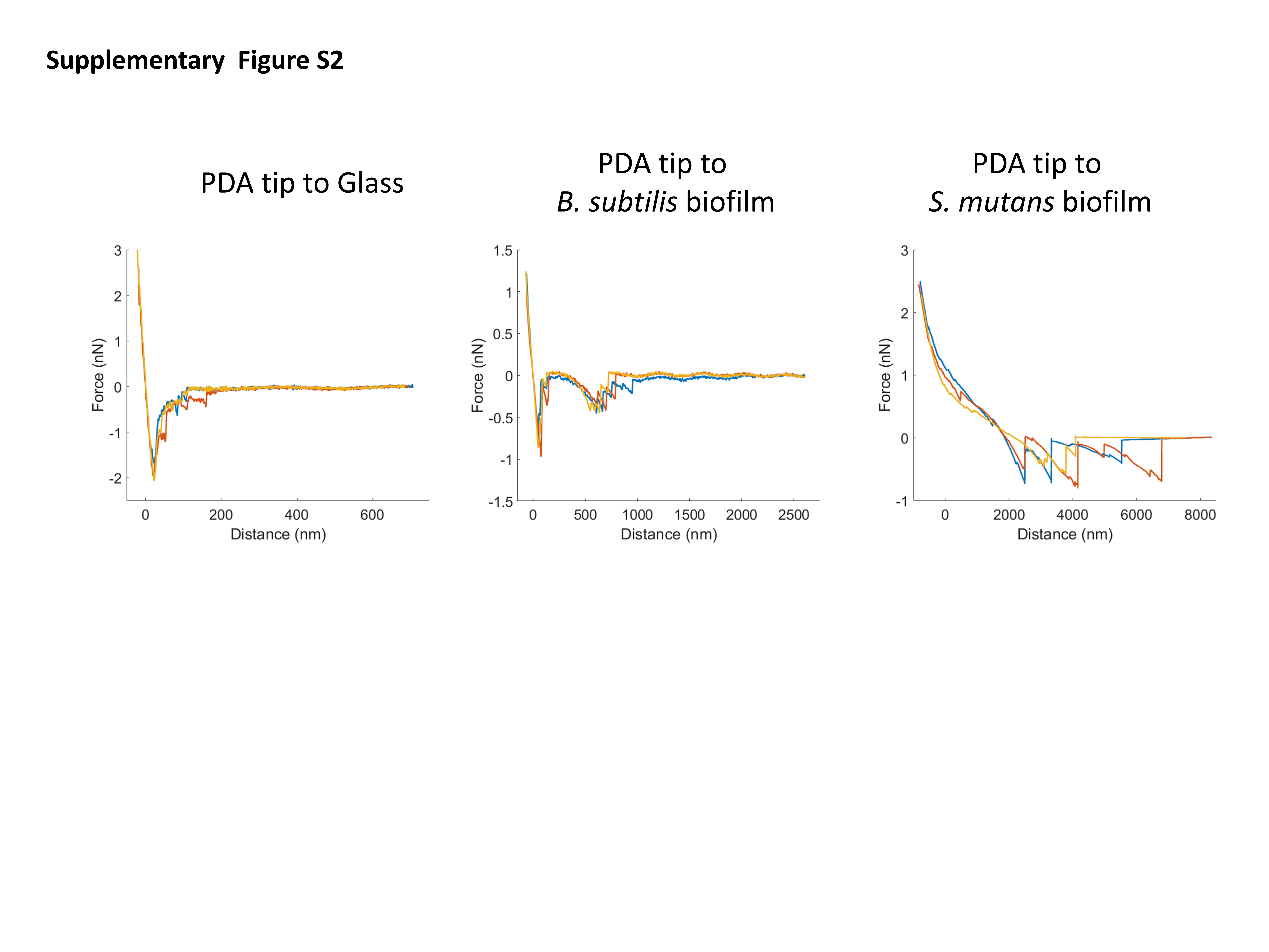


Supplementary Figure S2. representative force profiles of PDA tip on a glass surface, *B. subtilis* biofilm and *S. mutans* biofilm.
